# [^68^Ga]Ga-FAPI-04 PET MRI/CT in the evaluation of gastric carcinomas compared with [^18^F]-FDG PET MRI/CT: a meta-analysis

**DOI:** 10.1186/s40001-023-00997-9

**Published:** 2023-01-18

**Authors:** Yawen Wang, Wenhao Luo, Ye Li

**Affiliations:** 1 Eight-Year Medical Doctor Program, Chinese Academy of Medical Sciences & Peking Union Medical College, Beijing, 100730 China; 2 Department of General Surgery, Peking Union Medical College Hospital, Chinese Academy of Medical Sciences and Peking Union Medical College, PUMCH, 9 Dongdan 3rd Alley, Beijing, 100730 China; 3 Peking Union Medical College Hospital, Chinese Academy of Medical Sciences and Peking Union Medical College, PUMCH, 9 Dongdan 3rd Alley, Beijing, 100730 China

**Keywords:** [^68^Ga]Ga-FAPI-04, Peritoneal involvement, [^18^F]-FDG, Sensitivity, Gastric cancer

## Abstract

**Objectives:**

To compare the detection rates of [^68^Ga]Ga-FAPI-04 PET MRI/CT vs. [^18^F]-FDG PET MRI/CT in gastric cancer.

**Methods:**

An extensive librarian-led literature search of PubMed, Embase, Web of Science, the Cochrane Central Library, and ClinicalTrials.gov was performed. The primary outcomes were sensitivity in patient-based evaluations, detection of lymph node metastases, and peritoneal involvement.

**Results:**

Five studies, including 148 participants, were analyzed. [^68^Ga]Ga-FAPI-04 PET MRI/CT has a comparatively high sensitivity in patient-based evaluations compared with [^18^F]-FDG PET MRI/CT (risk difference = 0.16, 95% CI 0.09–0.22, *P* < 0.00001). The [^68^Ga]Ga-FAPI-04 PET MRI/CT group has a comparatively higher sensitivity in detecting lymph node metastases (RR = 0.15, 95% CI 0.01–0.29, *P* = 0.04), peritoneal involvement (RR = 0.55, 95% CI 0.38–0.72, *P* < 0.00001) in gastric cancer than [^18^F]-FDG PET MRI/CT group.

**Conclusions:**

This systematic review confirmed the advantage of [^68^Ga]Ga-FAPI-04 PET MRI/CT in gastric cancer. [^68^Ga]Ga-FAPI-04 PET MRI/CT was superior to [^18^F]-FDG PET MRI/CT in detecting the primary tumor, lymph node metastases, and peritoneal metastases. More studies are needed for the sensitivity and specificity of [^68^Ga]Ga-FAPI-04 PET MRI/CT in different pathological types of gastric cancer.

## Introduction

Gastric cancer is a vital cancer burden globally, with the fifth-highest diagnostic rate and the third-highest mortality rate [1]. Surgery or endoscopic resection is the primary treatment for gastric cancer. An early and accurate diagnosis of gastric cancer has a significant impact on the prognosis. Other prognostic factors include tumor stage, lymph node metastasis, pathological type, and adjuvant therapy [2].

Traditional [^18^F]-FDG PET MRI/CT is based on the enrichment of glucose tracers and is related to metabolic activities [3]. However, it has a considerable intake in normal tissues, such as the brain, liver and intestine, and has limited use in low metabolic tumors, such as prostate cancer. The detection rate of [^18^F]-FDG PET MRI/CT in gastric cancer is affected by pathological tumor type and high FDG uptake in the gastric wall, and the detection sensitivity is not ideal [4].

Fibroblast activating protein (FAP) is a type II transmembrane protease with dipeptidyl peptidase and endopeptidase activities [5]. It mainly exists in activated fibroblasts of cancer, chronic inflammation and fibrosis, and participates in tissue remodeling, angiogenesis and collagen degradation. FAP inhibitor (Fapi) is a radiolabeled quinoline tracer suitable for positron emission tomography (PET) [6]. [^68^Ga]Ga-FAPI-04 PET is highly expressed in various cancer types, including cancers with low [^18^F]-FDG affinity [7]. Moreover, the uptake of [^68^Ga]Ga-FAPI-04 PET in almost all normal tissues (including the brain and intestine) was low. Recent studies have shown that [^68^Ga]Ga-FAPI-04 PET can provide prognostic information, guide treatment choices, and help predict tumor invasiveness [7, 8]. However, the diagnostic value of [^68^Ga]Ga-FAPI-04 PET MRI/CT in gastric cancer remains to be studied.

This article searched the comparative studies comparing [^68^Ga]Ga-FAPI-04 PET MRI/CT and [^18^F]-FDG PET MRI/CT in gastric cancer. We discussed the difference in the detection rate of the primary tumor, lymph node metastasis, and peritoneal cancer metastasis. This article aims to provide a more optimized choice for the screening, condition evaluation and treatment effect monitoring of gastric cancer, and further improve the survival benefit of patients.

## Materials and methods

This systematic review was based on the Preferred Reporting Items for Systematic Reviews and Meta-analysis (PRISMA) statements.

### Study selections

The related studies were retrieved in the following databases: PubMed, Embase, Web of Science, the Cochrane Central Library, and ClinicalTrials.gov until 1st July 2022. For all databases, the search strategy includes the use of the following terms: “^68^Ga-FAPI-04 PET,” “^18^F-FDG PET” “Gastric cancer” “prospective studies”, “clinical trial”, and “randomized/randomized controlled study”. The language was limited to English. The retrieval was limited to comparative studies (trials). This meta-analysis was in line with the Critical Appraisal Skills Programme Checklist. Data extraction and conformity assessment were conducted by two independent reviewers (L.W.H. and W.Y.W). The differences among the reviewers were resolved through group discussion.

### Inclusion and exclusion criteria

Two independent reviewers (L.W.H. and W.Y.W.) assessed eligibility and reached a consensus by discussing differences with a third investigator (L.Y.). The evaluation was repeated twice. First of all, the title and abstract were preliminarily evaluated, and the full text was evaluated after the potentially qualified study was selected. No reviewers were blinded to the authors of these studies.

#### Inclusion criteria

1. Type of study: the analysis included only comparative trials published in fully peer-reviewed journals before 1st July 2022.
2. Language: only English articles were included.
3. Type of intervention: two different diagnostic techniques for gastric cancer, were assessed for diagnostic sensitivity of both the primary tumor and metastasis.
4. Type of participants: patients who developed gastric cancer were the target population for the meta-analysis.

#### Exclusion criteria

1. Non-comparative trials and unpublished studies were excluded.
2. No relevant results were found.

### Outcomes of interest

The primary outcome measure included (1) sensitivity in patient-based evaluations; (2) sensitivity in detecting lymph node metastases and (3) sensitivity in detecting peritoneal involvement.

### Data collection

We extracted the following data: first author, year of the study, country of origin, number of participants, type of diagnostic techniques, population characteristics, and all the relevant outcomes. Two authors (L.W.H. and W.Y.W) independently extracted and cross-checked all data. The differences were resolved through in-depth discussions with a third reviewer (L.Y.) until we reached a consensus.

### Evaluation of quality of evidence

Two independent reviewers (L.W.H. and W.Y.W) blindly evaluated the methodological quality of the selected studies. Differences were discussed among the groups and resolved by a third evaluator. The quality was evaluated using the CASP Checklist, which assesses the risk of bias, including 11 assessment items. Each study was assigned a score from 0 to 11. According to randomization, blinding, method quality and statistical reporting, 0 was the lowest quality and 11 was the highest.

Differences were settled by consensus.

### Statistical analysis

The data were entered into the Cochrane Collaboration’s Review Manager program (RevMan version 5.4; Cochrane Collaboration, Oxford, UK). For continuous data, the average and deviation of each study were required. We analyzed the risk variance with 95% confidence intervals (CIs) and tested the heterogeneity (*I*^2^ index) of the results. We used fixed-effects or random-effects models to combine summary data accordingly. We tested the publication bias with funnel plots. This paper reports the P values of the hypotheses test for the research variables. The effect was considered statistically significant if the *P* value was ≤ 0.05%.

## Results

### Literature search

**Fig. 1:**
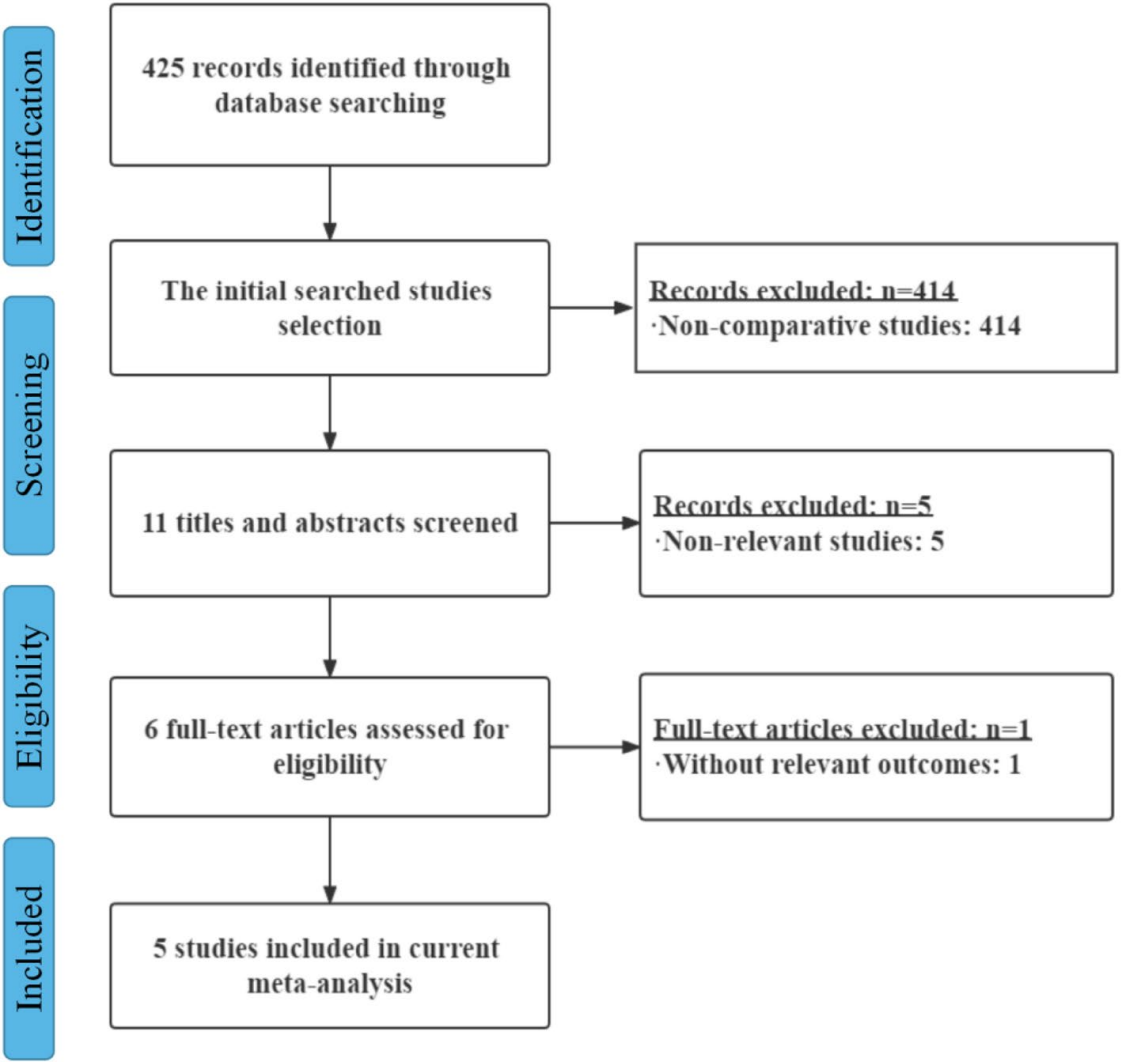
Flow diagram of the literature search

The flow diagram of literature retrieval is shown in Fig. 1. We screened out 425 articles that might meet the criteria. Five of them fulfilled the inclusion criteria. Initially, through an electronic database search, we identified 425 citations. The review of the list of references in all relevant papers, recent editorials and related review articles did not produce further evaluation research. Non-comparative studies were excluded, and the remaining 11 articles were selected after reading titles and abstracts. After carefully reading the full text of 11 articles, 5 studies were excluded, because the subjects had nothing to do with gastric cancer. One of the studies was further excluded due to the lack of relevant results. The other five studies were eventually incorporated into the qualitative analysis and final meta-analysis.

### Study characteristics

The characteristics of the selected study are shown in Table 1. Our meta-analysis included 148 patients. Among them, all patients were diagnosed with both [^68^Ga]Ga-FAPI-04 PET MRI/CT and [^18^F]-FDG PET MRI/CT. The quality assessment results included in the study are shown in Table 2.

**Table 1:** Baseline information of comparative studies enrolled in the meta-analysis

References	Country	Year	Design	Patients	Imaging examinations		Age	No. of patients
Qin et al.	China	2022	Comparative study	Histopathologically proven diagnosis of gastric cancer	^68^Ga-FAPI-04 MRI	^18^F-FDG CT	Median age 56 (range 29–70)	20 (9 m;11f)
Gündoğan et al.	Turkey	2021	Comparative study	Histopathologically proven diagnosis of gastric cancer	^68^Ga-FAPI-04 CT	^18^F-FDG CT	Median age 61 (range 40–81)	21 (12 m;9f)
Jiang et al.	China	2021	Comparative study	Histopathologically proven diagnosis of gastric cancer	^68^Ga-FAPI-04 MRI/CT	^18^F-FDG MRI/CT	Median age 67.5 (range 25–86)	38 (29 m,9f)
Lin et al.	China	2022	Comparative study	Histopathologically proven diagnosis of gastric cancer	^68^Ga-FAPI-04 CT	^18^F-FDG CT	Median age 63.8 ± 14.9 (range 28–85)	56 (40 m;16f)
Kuten et al.	Israel	2021	Comparative study	Histopathologically proven diagnosis of gastric cancer	^68^Ga-FAPI-04 CT	^18^F-FDG CT	Median age 70 (range 35–87)	13 (6 M,7F)

**Table 2:** Quality evaluations of comparative studies finally included in the meta-analysis

References	Score of item I	Score of item II	Score of item III	Score of item IV	Score of item V	Score of item VI	Score of item VII	Score of item VIII	Score of item IX	Score of item X	Score of item XI	Total scores
Qin et al.	0	1	0	1	1	0	1	1	1	1	1	8
Gündoğan et al.	0	1	0	1	1	0	1	1	1	1	1	8
Jiang et al.	0	1	0	1	1	0	1	1	1	1	1	8
Lin et al.	0	1	0	1	1	0	1	1	1	1	1	8
Kuten et al.	0	1	0	1	1	0	1	1	1	1	1	8

### Primary outcomes

### Sensitivity in patient-based evaluations

**Fig. 2:**
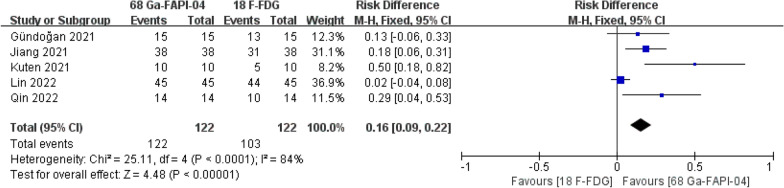
Forest plot of sensitivity in patient-based evaluations

Five of the included studies reported sensitivity in patient-based evaluations. [^68^Ga]Ga-FAPI-04 PET MRI/CT has a comparatively high sensitivity in patient-based evaluations compared with [^18^F]-FDG PET MRI/CT. (risk difference = 0.16, 95% CI 0.09–0.22, *P* < 0.01) (Fig. 2).

### Sensitivity in detecting lymph node metastases

**Fig. 3:**
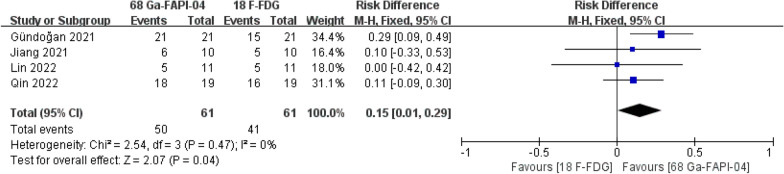
Forest plot of sensitivity in detecting lymph node metastases

Four studies reported sensitivity in detecting lymph node metastases. [^68^Ga]Ga-FAPI-04 PET MRI/CT was significantly better than [^18^F]-FDG PET MRI/CT in sensitivity in detecting lymph node metastases (risk difference = 0.15, 95% CI 0.01–0.29, *P* = 0.04) (Fig. 3).

### Sensitivity in detecting peritoneal involvement

**Fig. 4:**
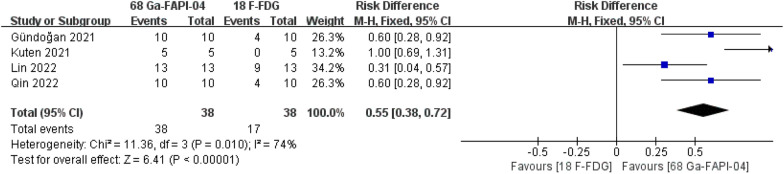
Forest plot of sensitivity in detecting peritoneal involvement

The sensitivity in detecting peritoneal involvement was reported in 4 studies. The sensitivity in detecting peritoneal involvement was 100% vs. 44.7% in [^68^Ga]Ga-FAPI-04 PET MRI/CT and [^18^F]-FDG PET MRI/CT, respectively. There was a significant difference in the sensitivity in detecting peritoneal involvement between [^68^Ga]Ga-FAPI-04 PET MRI/CT and [^18^F]-FDG PET MRI/CT. (risk difference = 0.55, 95% CI 0.38–0.72, *P* < 0.01) (Fig. 4).

### Potential publication bias

**Fig. 5:**
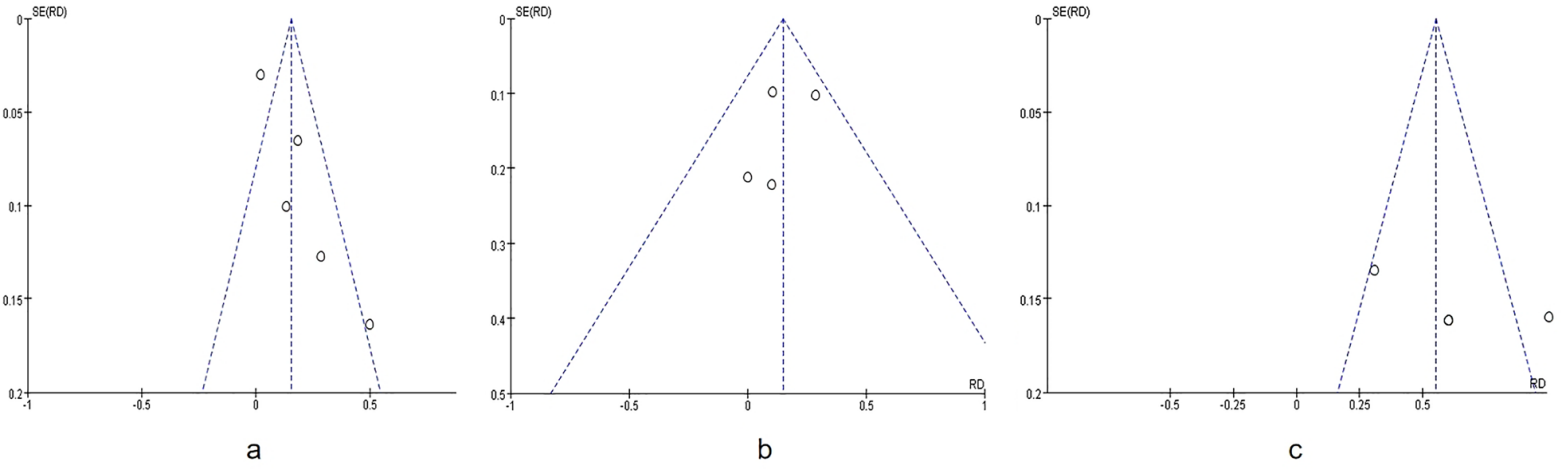
Funnel plot of the sensitivity in **a** patient-based evaluations; **b** detecting lymph node metastases and **c** detecting peritoneal involvement

A funnel plot regarding the sensitivity in patient-based evaluations; sensitivity in detecting lymph node metastases and sensitivity in detecting peritoneal involvement are demonstrated in Fig. 5, respectively. The funnel plot did not show obvious asymmetry. Since all studies were limited to other events, no significant publication bias was found.

## Discussion

This is the first systematic review and meta-analysis of all relevant comparative studies to compare the detection rates of [^68^Ga]Ga-FAPI-04 PET MRI/CT and [^18^F]-FDG PET MRI/CT in gastric cancer. This study included 148 participants from five independent comparative trials [9–13]. All participants had pathologically proven gastric cancer and received [^68^Ga]Ga-FAPI-04 PET MRI/CT and [^18^F]-FDG PET MRI/CT. As mentioned above, [^68^Ga]Ga-FAPI-04 PET MRI/CT was superior to [^18^F]-FDG PET MRI/CT in detecting the primary tumor [100.00% (122/122) vs. 84.43% (103/122)], lymph node metastases [81.97% (50/61) vs. 67.21% (41/61)] and peritoneal metastases [100.00% (38/38) vs. 44.74% (17/38)].

Our study found that [^68^Ga]Ga-FAPI-04 PET MRI/CT was significantly better than [^18^F]-FDG PET MRI/CT in detecting primary lesions of gastric cancer. Previous studies have shown that [^18^F]-FDG PET MRI/CT has limitations in diagnosing gastric cancer. Mukai et al. reported that the detection rate of [^18^F]-FDG PET in gastric cancer with tumor size less than 30 mm was 16.8%, and that in early gastric cancer was 25.9% [14]. One article we included found that the detection rate of [^68^Ga]Ga-FAPI-04 PET was higher than that of [^18^F]-FDG PET in tumors less than 4 cm (100% vs. 71%) [10]. The level of FDG uptake in gastric cancer is affected by pathological tumor types and physiological uptake of FDG by the gastric wall may also interfere with detection [15]. Two recent studies found that the detection rate of [^68^Ga]Ga-FAPI-04 PET in signet ring cell carcinoma was significantly higher than that of [^18^F]-FDG PET [10, 16]. The low level of FDG uptake in certain types of gastric cancer (mucinous, signet ring cell, and diffuse gastric adenocarcinoma) may be related to the diffuse infiltration of tumor cells and the increase of inert mucus [15, 17].

In gastric cancer studies, [^68^Ga]Ga-FAPI-04 PET MRI/CT not only has higher diagnostic sensitivity, but also has higher tracer uptake and TBR than [^18^F]-FDG PET MRI/CT [9–13, 16]. This may be related to the low physiological uptake of [^68^Ga]Ga-FAPI-04 PET in the stomach. Recent studies have found that the average SUV max of [^68^Ga]Ga-FAPI-04 PET in T2-4 tumors is significantly higher than that in T1 tumors (9.7 ± 4.4 vs. 3.1 ± 1.5), which provides a possibility for noninvasive judgment of the degree of invasion of gastric cancer [10]. Due to the high degree of malignancy of gastric cancer, early and accurate tumor identification significantly impacts treatment and prognosis. Therefore, [^68^Ga]Ga-FAPI-04 PET scan has potential for gastric cancer staging and can be use as complementary with [^18^F]-FDG PET scan.

Radical resection of gastric cancer requires complete resection of the primary tumor and removal of metastatic lymph nodes. Standard gastrectomy involves the removal of at least 2/3 of the stomach and the dissection of D2 lymph nodes (lymph nodes around the abdomen, the celiac axis, and the splenic artery) [18]. Lymph node staging will affect the scope of lymph node dissection and surgical methods, and impact the prognosis of patients [19]. Previous studies have shown that [^18^F]-FDG PET is less sensitive in detecting lymph node metastasis of gastrointestinal tumors [20]. Our study found that the sensitivity of [^68^Ga]Ga-FAPI-04 PET MRI/CT to lymph node metastasis was significantly higher than that of [^18^F]-FDG PET MRI/CT (81.97% vs. 67.21%). This may be related to the fact that lymph nodes are usually composed of fibroblast reticular cells, which are easier to be detected by fapi. To improve the detection rate of N2 or N3 lymph node metastasis by PET–CT and highlight the specific areas of high metabolic lymph nodes can optimize the surgical decision and treatment plan [15]. Therefore, the use of [^68^Ga]Ga-FAPI-04 PET MRI/CT in detecting gastric cancer is more helpful in accurately guiding clinical treatment.

Peritoneal carcinoma is common in gastrointestinal tumor metastasis. The extent of its involvement will determine the resectability and healing of the tumor, and further determine the prognosis. Due to the low level of FDG uptake in peritoneal carcinoma, the detection rate of [^18^F]-FDG in peritoneal carcinoma is poor, and it is easy to underestimate the degree of peritoneal involvement [21]. Our study found that [^68^Ga]Ga-FAPI-04 PET MRI/CT was highly sensitive to peritoneal metastasis of gastric cancer (100%). This may be due to the fibrotic reaction of tumor cells invading the peritoneum, and the target of fapi is fibroblast activating protein (FAP). Improving the detection rate of peritoneal carcinoma is helpful to more accurately judge the extent of disease involvement and evaluate the treatment response.

The common distant metastasis of gastric cancer includes the liver, lung, adrenal gland, bone, and ovary [22]. At present, there are few reports on the detection rate of [^68^Ga]Ga-FAPI-04 PET in distant metastasis of gastric cancer. Among the five comparative studies, two compared the detection rates of [^68^Ga]Ga-FAPI-04 PET and [^18^F]-FDG PET in patients with gastric cancer with ovarian, liver and bone metastases, and found no significant difference [12, 13].

To our knowledge, this is the first meta-analysis comparing [^68^Ga]Ga-FAPI-04 PET and [^18^F]-FDG PET in gastric cancer. Our study has some limitations. First, there are limited articles to evaluate the metastasis of other organs of gastric cancer. We cannot comprehensively and systematically assess the diagnostic value of [^68^Ga]Ga-FAPI-04 PET MRI/CT and [^18^F]-FDG PET MRI/CT. Second, the detection methods are not unified. PET CT was used in three articles [9, 11, 12], while PET MRI and PET CT were used in two articles [10, 13]. Finally, due to the low sample size and limited histopathological types of gastric cancer, it is impossible to compare the detection rates of [^68^Ga]Ga-FAPI-04 PET MRI/CT and [^18^F]-FDG PET MRI/CT according to pathological classification. However, each coin has two side. [^68^Ga]Ga-FAPI-04 PET has some limitations. Because of its high physiological uptake in the uterus and ovary, the detection of uterus or ovary metastasis may not be ideal. However, due to the limited number of patients with uterine or ovarian metastasis in the included cases, further systematic assessment was not possible.

In conclusion, this systematic review and meta-analysis confirmed that [^68^Ga]Ga-FAPI-04 PET MRI/CT had a higher detection rate of primary gastric cancer, peritoneal metastasis and lymph node metastasis than [^18^F]-FDG PET MRI/CT. 68Ga-FAPI-04 PET provides a possibility for noninvasive determination. The above conclusions need to be confirmed in more extensive cohort studies. More studies are required to explore the role of [^68^Ga]Ga-FAPI-04 PET MRI/CT in the prognosis of gastric cancer and its sensitivity and specificity in different pathological types of gastric cancer.

## Data Availability

Not applicable.

## References

[1] Sung H (2021). Global cancer statistics 2020: GLOBOCAN estimates of incidence and mortality worldwide for 36 cancers in 185 countries. CA: Cancer J Clin.

[2] Smyth EC (2020). Gastric cancer. Lancet.

[3] Salas JR, Clark PM (2022). Signaling pathways that drive F-FDG accumulation in cancer. J Nucl Medi.

[4] Jayaprakasam VS, Paroder V, Schöder H (2021). Variants and pitfalls in PET/CT imaging of gastrointestinal cancers. Semin Nucl Med.

[5] Zhao L (2022). Fibroblast activation protein-based theranostics in cancer research: a state-of-the-art review. Theranostics.

[6] Gilardi L (2022). Imaging cancer-associated fibroblasts (CAFs) with FAPi PET. Biomedicines.

[7] Sollini M (2021). State-of-the-art of FAPI-PET imaging: a systematic review and meta-analysis. Eur J Nucl Med Mol Imaging.

[8] Roustaei H (2022). Could fibroblast activation protein (FAP)-specific radioligands be considered as pan-tumor agents?. Contrast Media Mol Imaging.

[9] Gündoğan C (2022). Comparison of 18F-FDG PET/CT and 68Ga-FAPI-04 PET/CT in the staging and restaging of gastric adenocarcinoma. Nucl Med Commun.

[10] Jiang D (2022). Comparison of [Ga]Ga-FAPI-04 and [F]-FDG for the detection of primary and metastatic lesions in patients with gastric cancer: a bicentric retrospective study. Eur J Nucl Med Mol Imaging.

[11] Kuten J (2022). Head-to-head comparison of [Ga]Ga-FAPI-04 and [F]-FDG PET/CT in evaluating the extent of disease in gastric adenocarcinoma. Eur J Nucl Med Mol Imaging.

[12] Lin R (2022). [Ga]Ga-DOTA-FAPI-04 PET/CT in the evaluation of gastric cancer: comparison with [F]FDG PET/CT. Eur J Nucl Med Mol Imaging.

[13] Qin C (2022). Ga-DOTA-FAPI-04 PET/MR in the evaluation of gastric carcinomas: comparison with F-FDG PET/CT. J Nucl Med.

[14] Mukai K (2006). Usefulness of preoperative FDG-PET for detection of gastric cancer. Gastric Cancer: Off J Int Gastric Cancer Assoc Jpn Gastric Cancer Assoc.

[15] Akin EA (2020). Clinical impact of FDG PET/CT in alimentary tract malignancies: an updated review. Abdom Radiol.

[16] Pang Y (2021). Comparison of Ga-FAPI and F-FDG uptake in gastric, duodenal, and colorectal cancers. Radiology.

[17] Stahl A (2003). FDG PET imaging of locally advanced gastric carcinomas: correlation with endoscopic and histopathological findings. Eur J Nucl Med Mol Imaging.

[18] Japanese Gastric Cancer Association (2021). Japanese gastric cancer treatment guidelines 2018 (5th edition). Gastric Cancer: Off J Int Gastric Cancer Assoc Jpn Gastric Cancer Assoc.

[19] Lim JS (2006). CT and PET in stomach cancer: preoperative staging and monitoring of response to therapy. Radiographics.

[20] Findlay JM (2019). Routinely staging gastric cancer with F-FDG PET-CT detects additional metastases and predicts early recurrence and death after surgery. Eur Radiol.

[21] Sato H (2016). Factors affecting recurrence and prognosis after R0 resection for colorectal cancer with peritoneal metastasis. J Gastroenterol.

[22] Riihimäki M (2016). Metastatic spread in patients with gastric cancer. Oncotarget.

